# Vitamin A Supplementation in Early Life Enhances the Intestinal Immune Response of Rats with Gestational Vitamin A Deficiency by Increasing the Number of Immune Cells

**DOI:** 10.1371/journal.pone.0114934

**Published:** 2014-12-11

**Authors:** Xia Liu, Ting Cui, Yingying Li, Yuting Wang, Qinghong Wang, Xin Li, Yang Bi, Xiaoping Wei, Lan Liu, Tingyu Li, Jie Chen

**Affiliations:** 1 Children's Nutrition Research Center, Ministry of Education Key Laboratory of Child Development and Disorders, Key Laboratory of Pediatrics in Chongqing, CSTC2009 CA5002, Chongqing International Science and Technology Cooperation Center for Child Development and Disorders, Children's Hospital of Chongqing Medical University, Chongqing 400014, P.R. China; 2 Institute of Pediatrics, Children's Hospital of Chongqing Medical University, Chongqing 400014, P.R. China; 3 Laboratory of Intestinal Physiology and Pathology, Department of Surgery, University of Maryland School of Medicine, Baltimore, Maryland, United States of America; University of Pittsburgh, United States of America

## Abstract

Vitamin A is a critical micronutrient for regulating immunity in many organisms. Our previous study demonstrated that gestational or early-life vitamin A deficiency decreases the number of immune cells in offspring. The present study aims to test whether vitamin A supplementation can restore lymphocyte pools in vitamin A-deficient rats and thereby improve the function of their intestinal mucosa; furthermore, the study aimed to identify the best time frame for vitamin A supplementation. Vitamin A-deficient pregnant rats or their offspring were administered a low-dose of vitamin A daily for 7 days starting on gestational day 14 or postnatal day 1, day 14 or day 28. Serum retinol concentrations increased significantly in all four groups that received vitamin A supplementation, as determined by high-performance liquid chromatography. The intestinal levels of secretory immunoglobulin A and polymeric immunoglobulin receptor increased significantly with lipopolysaccharide challenge in the rats that received vitamin A supplementation starting on postnatal day 1. The rats in this group had higher numbers of CD8^+^ intestinal intraepithelial lymphocytes, CD11_C_^+^ dendritic cells in the Peyer's patches and CD4^+^CD25^+^ T cells in the spleen compared with the vitamin A-deficient rats; flow cytometric analysis also demonstrated that vitamin A supplementation decreased the number of B cells in the mesenteric lymph nodes. Additionally, vitamin A supplementation during late gestation increased the numbers of CD8^+^ intestinal intraepithelial lymphocytes and decreased the numbers of B lymphocytes in the mesenteric lymph nodes. However, no significant differences in lymphocyte levels were found between the rats in the other two vitamin A supplement groups and the vitamin A-deficient group. In conclusion, the best recovery of a subset of lymphocytes in the offspring of gestational vitamin A-deficient rats and the greatest improvement in the intestinal mucosal immune response are achieved when vitamin A supplementation occurs during the early postnatal period.

## Introduction

Many studies have reported that vitamin A (VA) acts as an anti-infection agent to reduce mortality [1] and morbidity in infants with diarrhea [2]. VA and its main metabolite, retinoic acid (RA), are required for the regulation of mucosal immunity and homeostasis. For instance, RA promotes the CD4^+^ T cell effector response via retinoic acid receptor alpha (RARα) [3] and modulates the differentiation of Foxp3^+^ regulatory T cells (Tregs) and Th17 effector T cells. RA also enhances the expression of polymeric immunoglobulin receptor (pIgR) [4] and the differentiation of IgA antibody-secreting cells (IgA-ASC) in mice and humans as well as stimulates the migration of T and B cells to the gut [5]–[9]. Our previous study demonstrated that VA deficiency (VAD) during gestation and early life altered the percentages of immune cells in the spleen and in gut-associated lymphoid tissues (GALT), including the mesenteric lymph nodes (MLNs) and Peyer's patches (PPs), and the numbers of intestinal intraepithelial lymphocytes (IELs) in the offspring, which may suppress the intestinal mucosal immune response [10]. These results prompted us to investigate the optimal time frame for VA supplementation (VAS).

It is well-established that VAS decreases mortality in children under the age of 5 years [11]. A meta-analysis has shown that VAS reduces the risks of diarrhea-related morbidity and mortality by 24% [12]. Our previous studies also indicated that supplementation with VA and multi-micronutrients or the combination of VA and eggs can increase physical growth and levels of hemoglobin and serum retinol in children [13]. Our survey and other studies [14], [15] have confirmed that children and pregnant women are at the highest risk of VAD-associated morbidity; VAD in pregnant women may lead to even more dangerous consequences in their offspring. However, the optimal time frame for VAS in pregnant women and younger children (especially newborns) with VAD remains controversial. In addition, it remains unclear whether VA intervention can mitigate the damage induced by VAD in subsets of lymphocytes and enhance the immune function of the intestinal mucosa.

In the current study, we used a rat model of gestational VAD to investigate the effective therapeutic window for VAS in the offspring of VAD rats. Based on the results of our previous study, VAS was provided on one of four days (gestational day 14 or postnatal days 1, 14 or 28) to determine which time point best activated the mucosal immune response against pathogens in the intestine. First, we detected the concentration of serum retinol and the level of fecal IgA in rat pups to confirm the effects of VAS in lipopolysaccharide (LPS)-infected rats. Second, we focused on the changes in various lymphocyte populations and their percentages in the spleen, mesenteric lymph nodes, Peyer's patches and intestinal intraepithelial lymphocytes in the four VAS groups in the presence or absence of LPS, with VAD rats as controls. Third, we examined the effect of VAS on pIgR expression in the intestine with or without LPS challenge using real-time PCR. Finally, we analysed these changes using a two-way analysis of variance (ANOVA) with the Bonferroni post hoc test to determine the main effects of VA supplementation and LPS challenge on changes in lymphocyte percentages. This study provides new information on the use of VA supplementation in early life as a successful strategy to prevent the mucosal immunosuppression caused by gestational VAD via the regulation of lymphocyte populations in the GALT.

## Materials and Methods

### Animals, diets and lipopolysaccharide challenge

Twenty SPF-grade female Wistar rats (6 weeks old) were obtained from the Experimental Animals Center of the Third Military Medical University (Chongqing, China). This study was conducted in strict accordance with the recommendations in the National Institutes of Health's Guide for the Care and Use of Laboratory Animals. The protocol was approved by the Animal Experimentation Ethical Committee of Chongqing Medical University (Chongqing, China). Two or three female rats per cage were in the same temperature-controlled (22–24°C) room with a constant airflow system and a 12-h light/dark cycle. All of the rats were fed VAD feed containing 400 IU/kg VA for 4 weeks to generate the maternal VAD animal model [16], [17]. Once the concentration of retinol in the serum of the VAD rats decreased to 1.05 µmol/L, the females were mated with normal males.

The offspring of the twenty female rats who had VAD during the gestational period were divided into five groups (n = 4; f. 1A, 1B). The pups of the gestational VAD rats that did not receive VA supplementation were fed VAD diets for 6 weeks after they were weaned (VAD group). Four different VAS time points were tested: gestational day 14 (VASP group) and postnatal day 1 (VAS1 group), day 14 (VAS2 group) and day 28 (VAS3 group). Daily VA doses of 50 IU, 63 IU, 75 IU and 100 IU were intragastrically administered for 7 days to the rats in the VAS1, VAS2, VAS3 and VASP groups, respectively [18], [19]. Following VA supplementation, the lactating female rats in the VASP, VAS1 and VAS2 groups were fed a VA-normal (VAN) diet (6500 IU VA/kg) until the weaning period. Following weaning, the pups were fed the VAN diet until they reached 6 weeks of age. The offspring in the VAS3 group were nursed by VAD maternal rats during the lactation period and fed the VAD diet until postnatal day 28; at that point, the offspring were given the VAN diet after VA supplementation until they reached 6 weeks of age.

**Figure 1:**
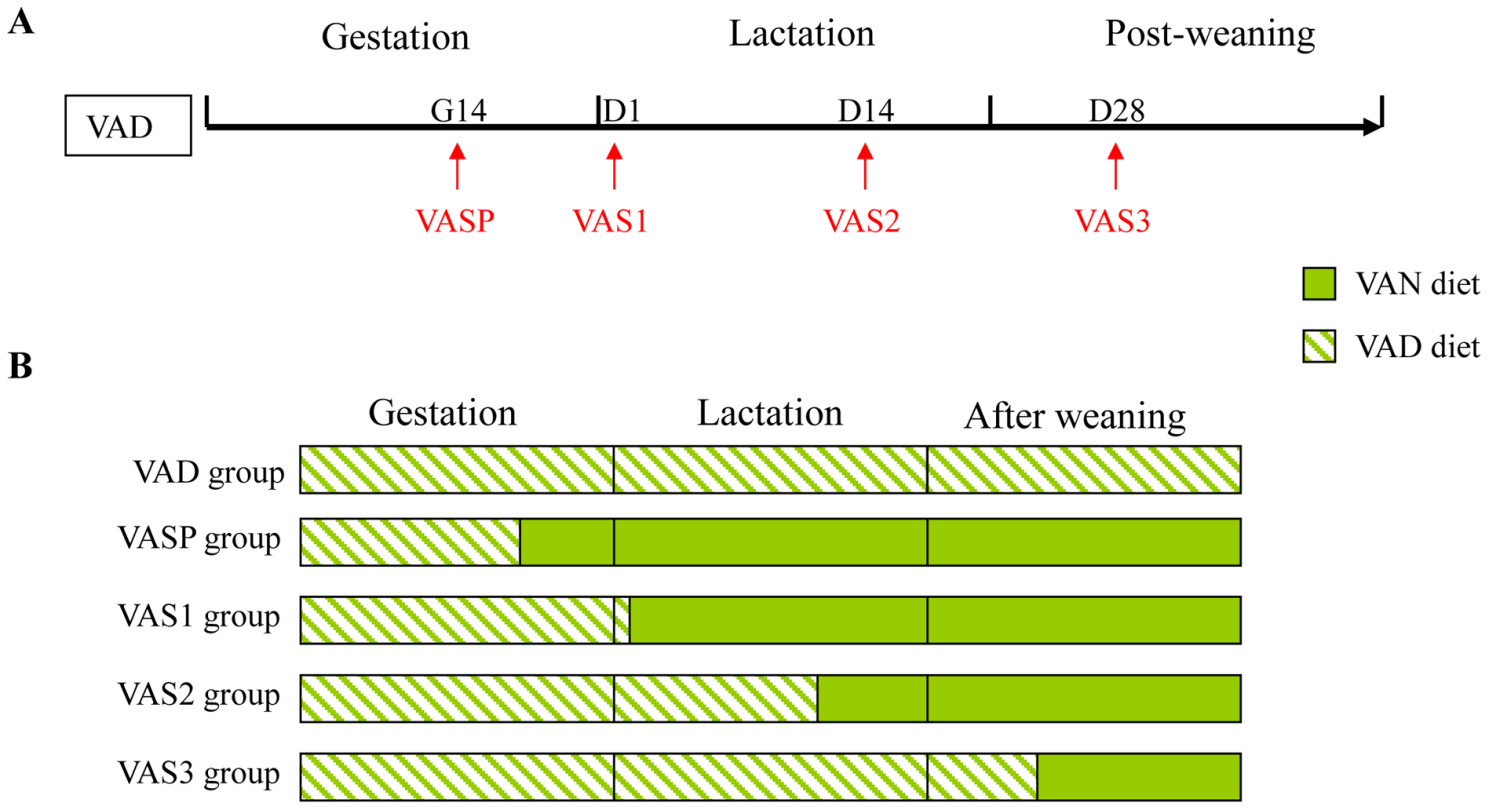
Schematic diagram showing the four time points at which VAS was provided (VASP, VAS1, VAS2 and VAS3). The rats in the VASP group were supplemented with VA beginning on gestational day 14 (G14). The lactating maternal rats were then fed a VAN diet followed by VAS until the weaning period; the pups of the rats in the VASP group were given the same VAN diet after they were weaned until they reached 6 weeks of age. The pups (VAS1 group) of gestational VAD rats were supplemented with VA on postnatal day 1 (D1), and the nursing maternal rats were fed a VAN diet until the weaning period. After weaning, the pups in the VAS1 group were given the same VAN diet until they reached 6 weeks of age. The pups (VAS2 group) of gestational VAD rats were supplemented with VA on postnatal day 14 (D14), and the nursing maternal rats were fed a VAN diet followed by VAS until the weaning period. After weaning, the pups in the VAS2 group were given the same VAN diet until they were 6 weeks old. The rats in the VAS3 group were supplemented with VA on postnatal day 28 (D28) and fed the VAN diet for 2 weeks following VAS.

For the LPS challenge, half of the 6-week-old pups in each group were randomly selected and injected intraperitoneally with 3 mg/kg of LPS from *E. coli* O111:B4; the other half of the pups were injected with an equal volume of PBS as the control. Following the 12-h challenge, the rats were sacrificed with urethane. Blood was immediately harvested from the rats's femoral arteries, and feces were collected from the ileum. The spleen, mesenteric lymph nodes and intestinal tissues were isolated and placed in 0.01 M PBS for future use.

### Serum retinol detection

Serum retinol concentrations were measured using high-performance liquid chromatography (HPLC) according to our previously described methods [20], with slight modifications. Briefly, 200 µl of serum was deproteinized with an equal volume of dehydrated alcohol. A total of 1,000 µl of hexane was used to extract the retinol from the serum; the hexane was then evaporated using nitrogen gas. The retinol residue was dissolved in 100 µl of the mobile phase mixture (methanol: water  = 97∶3). Finally, the prepared sample was measured using an HPLC apparatus (DGU-20As, Shimadzu Corporation, Kyoto, Japan) on a C18 analytical column with a 315-nm ultraviolet photodiode array detector.

### Detection of IgA in the feces

The concentration of secretory IgA (sIgA) in the intestine was measured using an ELISA kit (Bethyl Lab, Inc., USA) according to the manufacturer's instructions. Briefly, according to the method described by Frossard [21], 250 mg of feces obtained from the ileocecum of rats was resuspended and incubated in 1 ml of 0.01 M PBS containing 1% fetal bovine serum (FBS) and 0.1 mM phenylmethanesulfonyl fluoride (PMSF, Solarbio, Amresco) for 1 hr at room temperature. The mixture was vortexed for 2 min and centrifuged at 14,000 rpm for 20 min at 4°C to obtain the supernatant. The concentration of total sIgA in the supernatant was determined using an ELISA kit. The results were calculated with a standard curve and expressed as nanograms of sIgA per milliliter of supernatant for 250 mg of feces.

### Isolation of lymphocytes from the spleen and mesenteric lymph nodes

The spleen and MLNs tissues were cut into pieces with surgical scissors. To obtain a single-cell suspension, these pieces of tissue were passed through a 200-mesh metallic grid with PBS. The cells were washed twice with PBS, and approximately 3×10^7^ cells remained for the flow cytometry analysis.

### Preparation of the Peyer's patches

The PPs were carefully removed from the serosal side of the intestine. After the residual mucus of the PPs was removed with PBS containing 1% dithiothreitol, the PPs were shredded into small pieces with surgical scissors on a 300-mesh metallic grid. Then, 2 ml of FBS was used to release single cells from the PPs, and the cell suspension was carefully added to the surface of a lymphocyte separation medium for rats (Tianjin Haoyang Biological Manufacture Co., China). Following centrifugation at 2,000 rpm for 15 min, the white flocculent substance on the interface between the FBS and the lymphocyte separation medium was transferred to a clean tube using a Pasteur pipette. The lymphocyte suspension was washed twice and resuspended with 100 µl of PBS for flow cytometry.

### Isolation of intestinal intraepithelial lymphocytes

The entire intestine was rinsed 3 times with 0.01 M PBS to remove the intestinal contents. After the PPs were removed from the intestine, the entire intestine was incubated in cold PBS with 2% FBS and 1% penicillin/streptomycin on ice for 2 hrs. Then, the entire intestine was washed twice with warmed Dulbecco's modified Eagle's medium (DMEM) containing 2% FBS and 1% dithiothreitol at 37°C to loosen the cells on the surface of the intestinal mucosa. Ophthalmic curve tweezers were used to gently squeeze the tissue along the length of the intestine to expel the cells of the mucosal layer. After the cell-DMEM mixture was incubated for 15 min at room temperature, the supernatant was collected and centrifuged at 2,000 rpm for 10 min. The pellet was resuspended with FBS, and the lymphocytes were isolated using the lymphocyte separation medium for rats (Tianjin Haoyang Biological Manufacture Co., China). The lymphocytes were washed twice with PBS for flow cytometry analysis.

### Flow cytometry

The monoclonal antibodies PECy5-anti-CD45, APC-anti-CD3, PE-anti-CD4, FITC-anti-CD8 and FITC-anti-CD25 were used to label the T lymphocytes, and PECy5-anti-CD45 and FITC-anti-CD45R (BD Pharmingen, San Diego, CA) were used to label the B lymphocytes. PECy5-anti-CD45 and PE-anti-CD11c were used as specific markers for dendritic cells (DCs), and the IELs were labeled using APC-anti-CD3, FITC-anti-CD8 and PE-anti-TCRγδ. Individual cell populations were identified according to the presence of specific fluorescence-labeled antibodies, and isotype-matched monoclonal antibodies were used as negative controls. All analyses were performed with an acquisition of at least 30,000 events on a BD FACSCanto II flow cytometer (Becton Dickinson).

### Real-time quantitative polymer chain reaction

The total RNA was extracted from the intestine using an RNA isolation kit (Bioteke Co., China) according to the manufacturer's instructions. First-strand cDNA was generated from the 1 µg of total RNA using the PrimeScript RT reagent kit (TaKaRa, China). The PCR templates were prepared with a 6-fold dilution of the first-strand cDNA product. The rat pIgR primer sequences were CCCCAGGATGTGAGTAGTATTG (forward) and CCTTGTCGGCACCAGTATTTC (reverse). Real-time PCR analysis was performed using a RealMasterMix SYBR green kit (TaKaRa), and the real-time PCR amplification protocol was performed with a Bio-Red real-time PCR instrument as follows: 95°C for 30 s; 39 cycles of 95°C for 5 s and 53.7°C for 30 s. The. primary data for the delta Ct values were normalized to the GAPDH values for the same sample to calculate the pIgR ratio.

### Statistics

All data are expressed as the mean±SD. The statistical analysis was performed using a two-way analysis of variance with the Bonferroni post hoc test (GraphPad Prism, version 5.0 software package). When there was a statistically significant interaction, all experimental groups were compared using the Bonferroni post hoc test. However, when there were no significant interactions, the main effects of VA or LPS were determined. Tukey's post hoc test was used to analyse the main effect of VA among the five combined VA treatment groups, and Student's t-test was used to analyse the primary effect of LPS in the two combined groups (i.e., those with and without LPS exposure) [22], [23]. *P* values less than 0.05 were considered statistically significant.

## Results

### Vitamin A supplementation increased serum retinol levels

**Figure 2:**
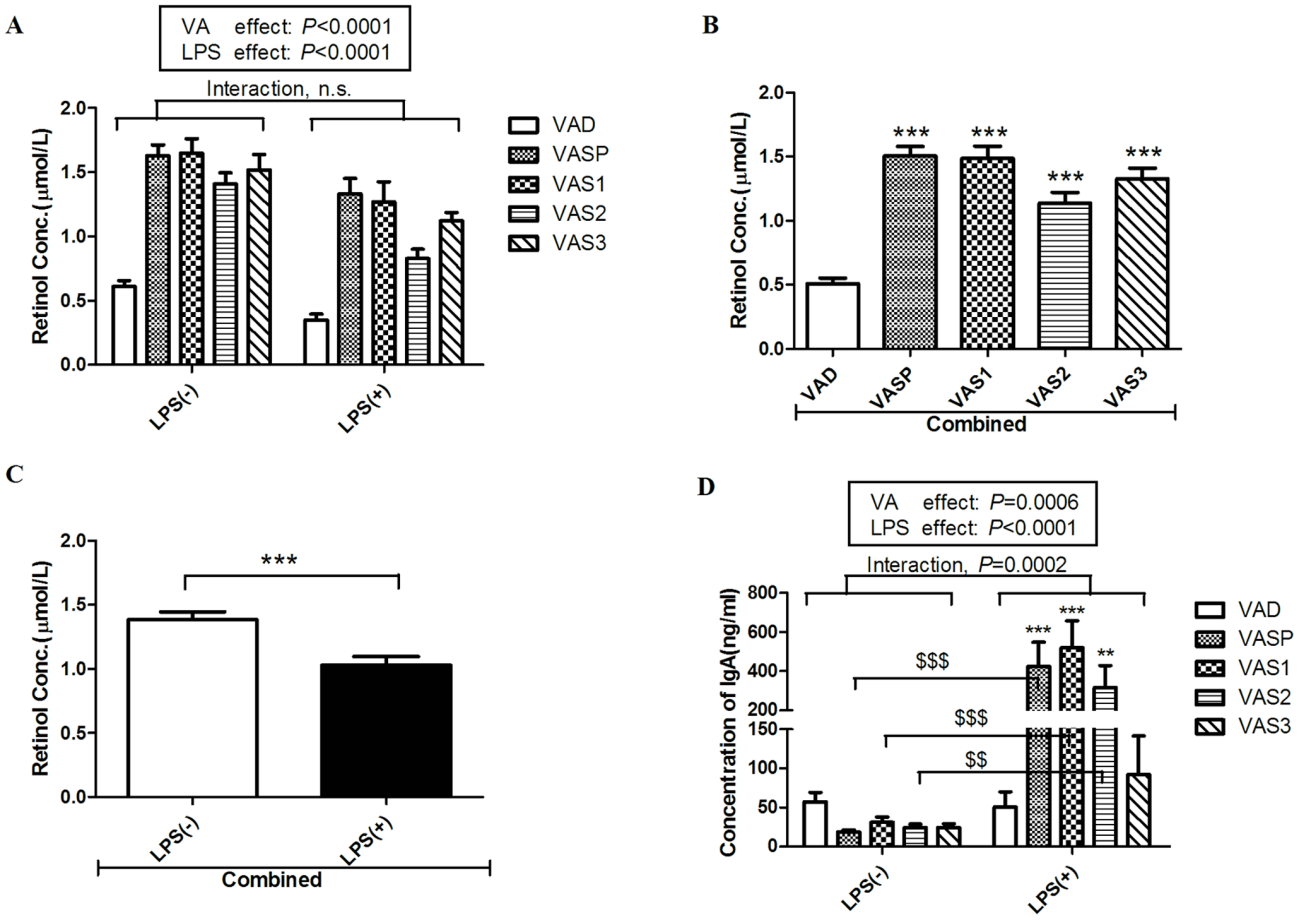
Effects of VAS and exposure to LPS on serum retinol levels and IgA concentrations in the intestinal stool of 6-week-old pups. A) Changes in the levels of serum retinol (µmol/L) in the rats in the five groups (VAD, VASP, VAS1, VAS2 and VAS3) in the presence or absence of LPS treatment (n = 10 to 16) according to HPLC analysis. B) VAS, independent of the LPS challenge, significantly increased the serum retinol concentrations (µmol/L) of the rats in the four combined VAS groups. C) The LPS treatment significantly decreased the serum retinol levels in the combined LPS group independent of VAS. D) Changes in sIgA in the intestinal stool of the rats in the five groups (VAD, VASP, VAS1, VAS2 and VAS3) with or without LPS challenge (n = 5). The values are the means±SD, **P<0.01, ***P<0.001, ^$$$^ *P*<0.001, ^$$^ *P*<0.01; n.s. = not significant in post hoc tests.

The maternal VAD animal model was produced in accordance with the international standard for VA levels in humans [24]. As Fig. 2A shows, the serum retinol concentration was 0.61±0.18 µmol/L in the offspring of VAD female rats, which suggests that VAD pups were successfully generated in the present study. Following supplementation, the serum retinol levels of the 6-week-old rats were significantly higher in the rats in the VASP, VAS1, VAS2 and VAS3 groups than in the rats in the VAD group with or without LPS challenge (Fig. 2A). Although the serum retinol levels decreased slightly following LPS challenge, there was no statistically significant interaction between the LPS and VA treatments. However, the Bonferroni post hoc test indicated that both the VA level and the LPS treatment significantly affected the serum retinol levels (*P*<0.0001). After the data from the LPS and the LPS-null treatment were combined, the retinol levels were significantly higher in the rats in the four VAS groups (VASP, VAS1, VAS2 and VAS3) than in the rats in the VAD group (*P*<0.001; Fig. 2B). The serum retinol levels were significantly lower in the LPS-challenged rats than in the rats in the combined LPS-null treatment group (*P*<0.001; Fig. 2C). These data demonstrate that VAS at any of the four time points could effectively increase the pups' serum retinol levels and that the intraperitoneal LPS challenge caused a significant decrease in serum retinol levels.

### Vitamin A supplementation enhanced intestinal IgA secretion following lipopolysaccharide challenge

sIgA participates in the intestinal innate immune response to protect the epithelium from enteric pathogens and toxins. Fig. 2D shows that the IgA concentrations in the intestinal feces were markedly increased in the rats in three groups (VASP, VAS1 and VAS2) that received LPS treatment; moreover, the IgA levels were significantly higher in the rats in these three groups that were challenged with LPS compared with the rats in the VAD+LPS group. The post hoc test showed that the VA level (*P* = 0.0006) and LPS treatment (*P*<0.0001) significantly affected IgA levels; moreover, the *P* value of the interaction between the LPS challenge and the VA level was 0.0002 (Fig. 2D). The above results suggest that VAS during the last stage of gestation or during early postnatal life can enhance intestinal IgA secretion following LPS challenge, which may improve mucosal immunity.

### Vitamin A supplementation on postnatal day 1 increased T lymphocyte populations in the spleen

**Figure 3:**
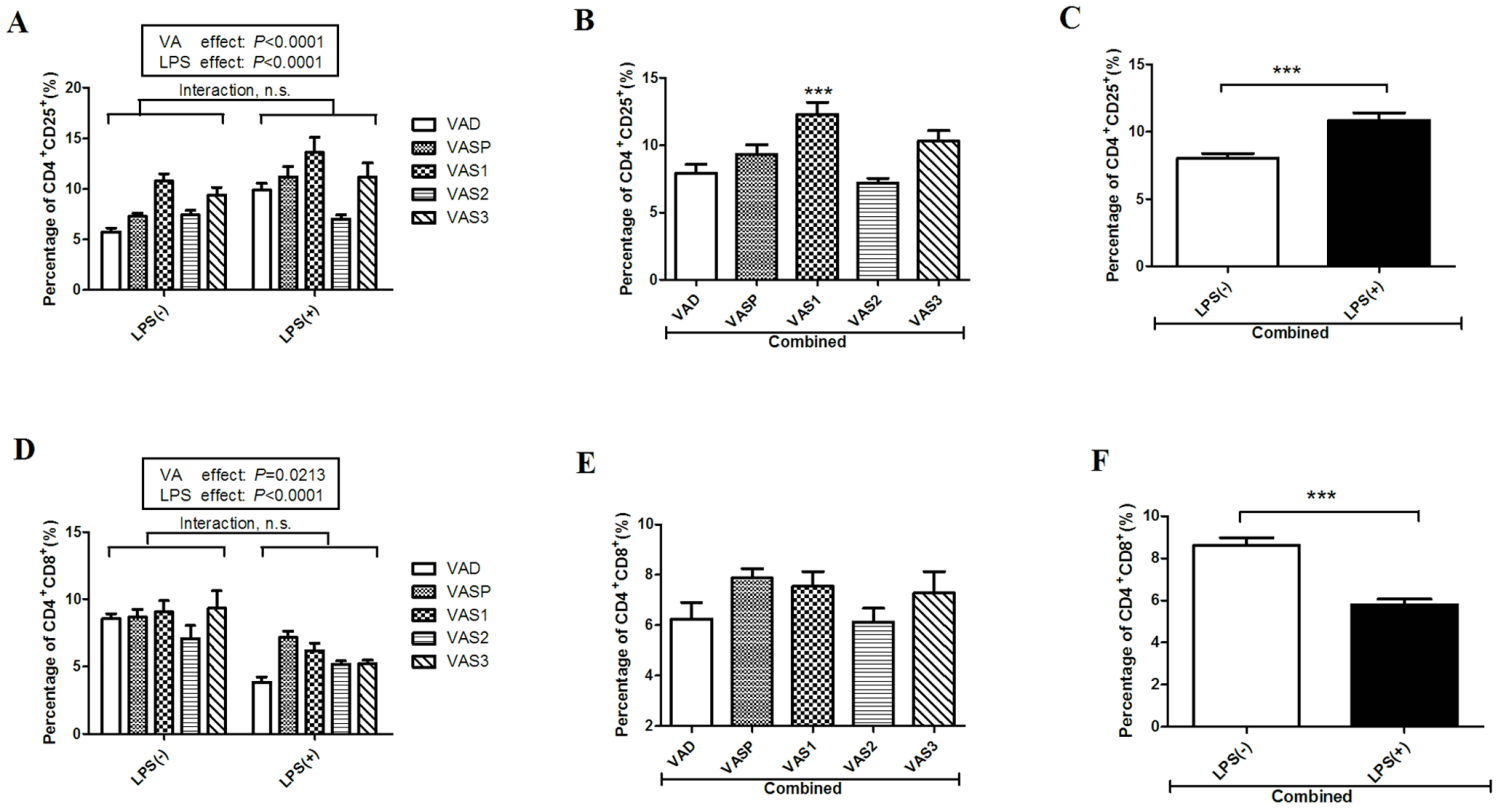
Changes in the percentages of CD4^+^CD25^+^ and CD4^+^CD8^+^ T lymphocytes in the spleens of rats in the VAD group and rats in the four groups (VASP, VAS1, VAS2 and VAS3) in the presence or absence of LPS. A) Effects of VAS and LPS challenge on the percentages of CD4^+^CD25^+^ T cells in the spleens of the rats in the five groups (n = 8 to 10). B) The percentage of CD4^+^CD25^+^ T cells was significantly higher in the rats in the combined VAS1 group compared with the rats in the combined VAD group. C) The LPS challenge significantly enhanced the number of CD4^+^CD25^+^ T cells after the five VA treatments were combined. D) The effects of VAS and LPS challenge on the percentages of CD4^+^CD8^+^ T cells in the spleens of the rats in the five groups (n = 8 to 10). E) There were no significant differences in the percentage of CD4^+^CD8^+^ T lymphocytes among the rats in the combined groups (VAD, VASP, VAS1, VAS2 and VAS3), although the *P* value of the VA effect was 0.0213. F) Exposure to LPS significantly reduced the number of CD4^+^CD8^+^ T lymphocytes after the five VA treatments were combined. The values are the means±SD, and ***P<0.001, n.s. = not significant in post hoc tests.

Our previous study demonstrated that rats with VAD in early life produced offspring with altered splenic T lymphocytes. To evaluate the effects of VAS at different time points, we used flow cytometry to compared the percentages of splenic T lymphocytes that were CD4^+^CD25^+^ T cells and CD4^+^CD8^+^ T cells in the rats in the four VAS groups and in the rats in the VAD group. As Fig. 3A shows, the percentage of CD4^+^CD25^+^ T lymphocytes was significantly higher in the rats in the VAS1 group (10.78±1.99%) compared with the rats in the VAD group (5.77±1.05%) in the absence of LPS challenge. LPS challenge led to a further increase in the percentage of CD4^+^CD25^+^ T lymphocytes in the rats in the VAD, VASP and VAS1 groups. The interaction between the VA level and LPS challenge did not significantly affect the percentage of CD4^+^CD25^+^ T cells. Additionally, both the LPS treatment (P<0.0001) and the VA intervention (P<0.0001) independently affected the percentage of CD4^+^CD25^+^ T cells in the spleen. The rats in the combined VAS1 group had significantly higher percentages of CD4^+^CD25^+^ T lymphocytes compared with the rats in the combined VAD group (Fig. 3B), whereas no differences in percentages were found among the rats in the VAD, VASP, VAS2 and VAS3 groups; this suggests that only VAS at postnatal day 1 is effective in mitigating the decrease in CD4^+^CD25^+^ T lymphocytes caused by gestational VAD. The data in Fig. 3C indicate that the percentage of CD4^+^CD25^+^ T cells was significantly increased in the rats in the combined LPS challenge group (*P*<0.001). Although the VA treatment significantly affected the percentage of CD4^+^CD8^+^ T cells in the spleen (*P* = 0.0213; Fig. 3D), there were no significant differences in the percentage of CD4^+^CD8^+^ T cells among the rats in the VAD, VASP, VAS1, VAS2 and VAS3 groups (Fig. 3E). However, in all of these groups, the rats that underwent the LPS challenge had significantly lower percentages of CD4^+^CD8^+^ T cells (Fig. 3F); this suggests that LPS challenge can effectively activate the systemic immune response by up-regulating the number of CD4^+^CD25^+^ T lymphocytes and down-regulating the number of CD4^+^CD8^+^ T cells.

### Vitamin A supplementation in early life decreased the percentage of B lymphocytes in the mesenteric lymph nodes

**Figure 4:**
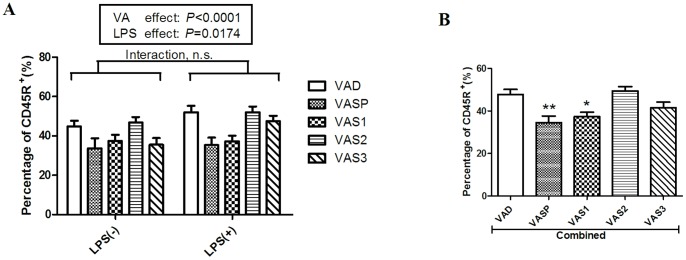
Effects of VAS and LPS challenge on the numbers of B lymphocytes in MLNs (n = 8 to 10). A) The post hoc analysis indicated that the *P* values of the effects of VA and LPS on the percentage of CD45R^+^ B cells were less than 0.0001 and 0.0174, respectively. B) The numbers of CD45R^+^ B cells were significantly lower in the rats in the combined VASP and VAS1 groups compared with the rats in the VAD group. The values are the means±SD, and *P<0.05, **P<0.01; n.s. = not significant in post hoc tests.

In our previous study, gestational VAD (independent of LPS treatment) resulted in an increase in the percentage of B lymphocytes in the MLNs and a further decrease in the migration of B lymphocytes to the intestine. As shown in Fig. 4A, VAS (*P*<0.0001) and LPS treatments (*P* = 0.0174) significantly affected the proportion of lymphocytes in the MLN that were B cells; however, two-way ANOVA found no statistical interaction between the effects of VA and LPS. After combining the data from the rats treated with LPS and the rats in the LPS-null group, we found that the percentages of B cells were significantly lower in the rats in the VASP and VAS1 groups than in the rats in the VAD group (Fig. 4B); however, there were no significant differences in percentages among the rats in the VAS2, VAS3 and VAD groups. Although the *P* value of the LPS effect on the percentage of B lymphocytes was 0.0174, no significant difference in this factor was detected between the rats challenged with LPS and the rats in the LPS-null treatment group (data not shown). The above findings indicate that VAS during the last stage of gestation and during the early postnatal stage can decrease the percentage of B lymphocytes in the MLNs independent of LPS challenge.

### Vitamin A supplementation on postnatal day 1 increased the number of dendritic cells in the Peyer's patches

**Figure 5:**
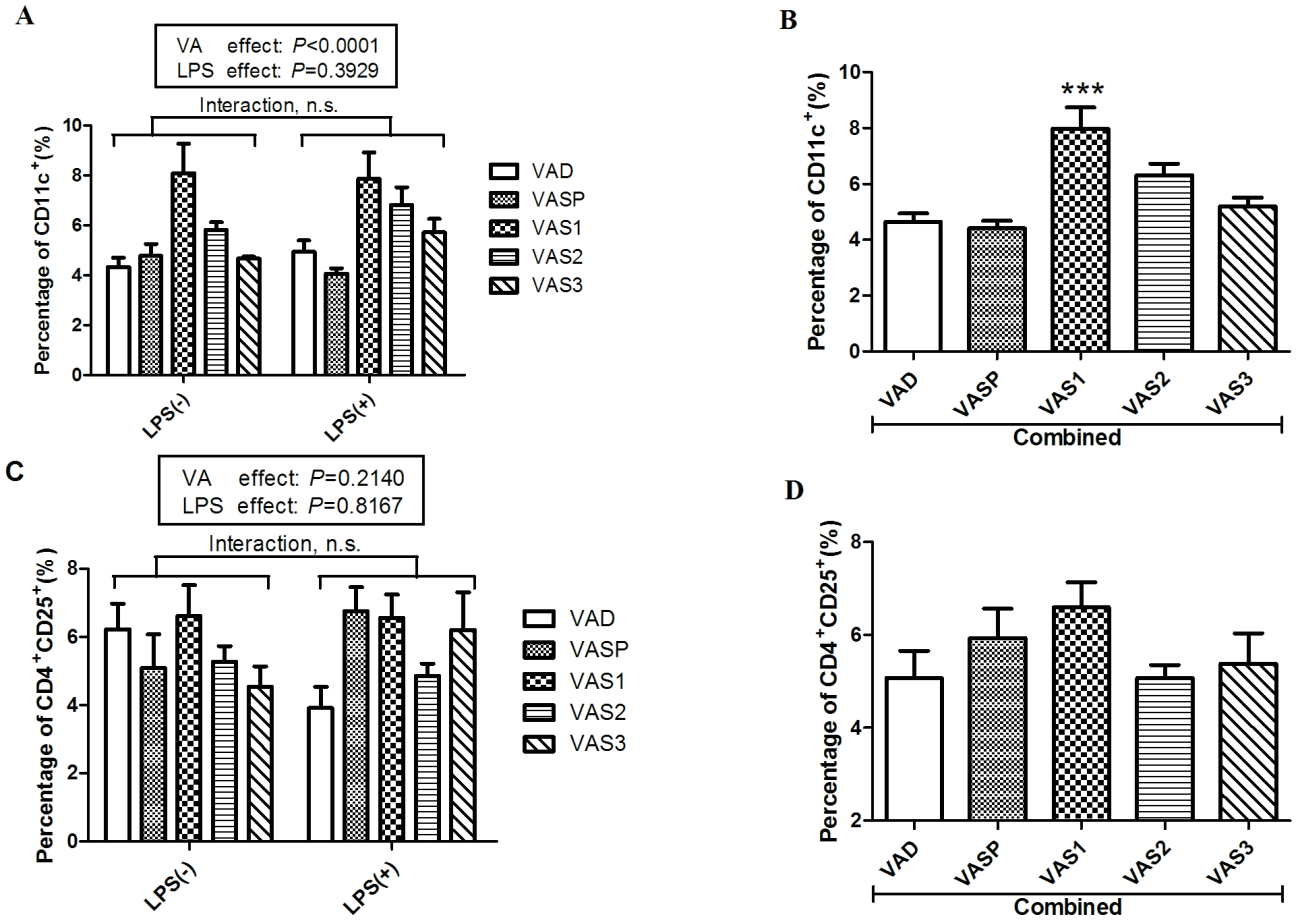
Changes in the number of CD11c^+^ DCs and CD4^+^CD25^+^ T cells in the Peyer's patches of rats in the VAD group and rats of the four VAS groups in the presence or absence of LPS (n = 16) . A) The effects of VAS and LPS treatment on the percentage of CD11c^+^DCs cells in the PPs of the rats in the five groups. B) The number of CD11c^+^DCs cells was significantly higher in the rats in the combined VAS1 group compared with the rats in the combined VAD group. C) The effects of VAS and LPS treatment on the percentage of CD4^+^CD25^+^ T cells in the PPs of the rats in the five groups. D) No differences were found in the percentage of CD4^+^CD25^+^ T cells between the rats in the combined VAD group and the rats in the four VAS groups. The values are the means±SD, and ***P<0.001; n.s. = not significant in post hoc tests.

PPs are inductive sites involved in the mucosal immune response of the intestine. Our previous study indicated that gestational VAD decreased the number of CD11c^+^ DCs and CD4^+^CD25^+^ T cells in the PPs. To investigate the effect of VAS on the protective immunity of the intestine, we measured the percentage of all lymphocytes that were CD11c^+^ DCs and the percentage of all T lymphocytes that were CD4^+^CD25^+^ T cells in the PPs of the rats in the five groups (VAD, VASP, VAS1, VAS2 and VAS3). A two-way ANOVA indicated that the interaction between VAS and LPS had no significant effects on the percentage of CD11c^+^ DCs (Fig. 5A). After combining data from the rats with LPS exposure and the rats without LPS exposure, we found that the number of CD11c^+^ DCs was significantly higher in the rats in the VAS1 group than in the rats in the VAD group (*P*<0.001; Fig. 5B). However, neither the VA (*P* = 0.2140) nor the LPS treatment (*P* = 0.8167) significantly affected the percentage of CD4^+^CD25^+^ T cells within the PPs (Fig. 5C). Additionally, no significant differences were found between the rats in the four combined VAS groups and the rats in the combined VAD group, although the numbers of CD4^+^CD25^+^ T cells tended to be higher in the pups in the combined VASP and VAS1 groups than in the pups in the combined VAD group (Fig. 5D). These results imply that VAS on postnatal day 1 can effectively increase the number of CD11c^+^ DCs in the PPs but not the number of CD4^+^CD25^+^ T cells. In the present study, both LPS challenge and VA supplementation failed to change the percentages of CD4^+^CD25^+^ T cells in the PPs, which was inconsistent with the results obtained for the splenic CD4^+^CD25^+^ T cells. This conflicting result may have occurred because LPS was administered via intraperitoneal injection and therefore elicited only a systemic immune response rather than an intestinal mucosal immune response.

### Vitamin A supplementation in early life increased the number of intestinal epithelial lymphocytes

**Figure 6:**
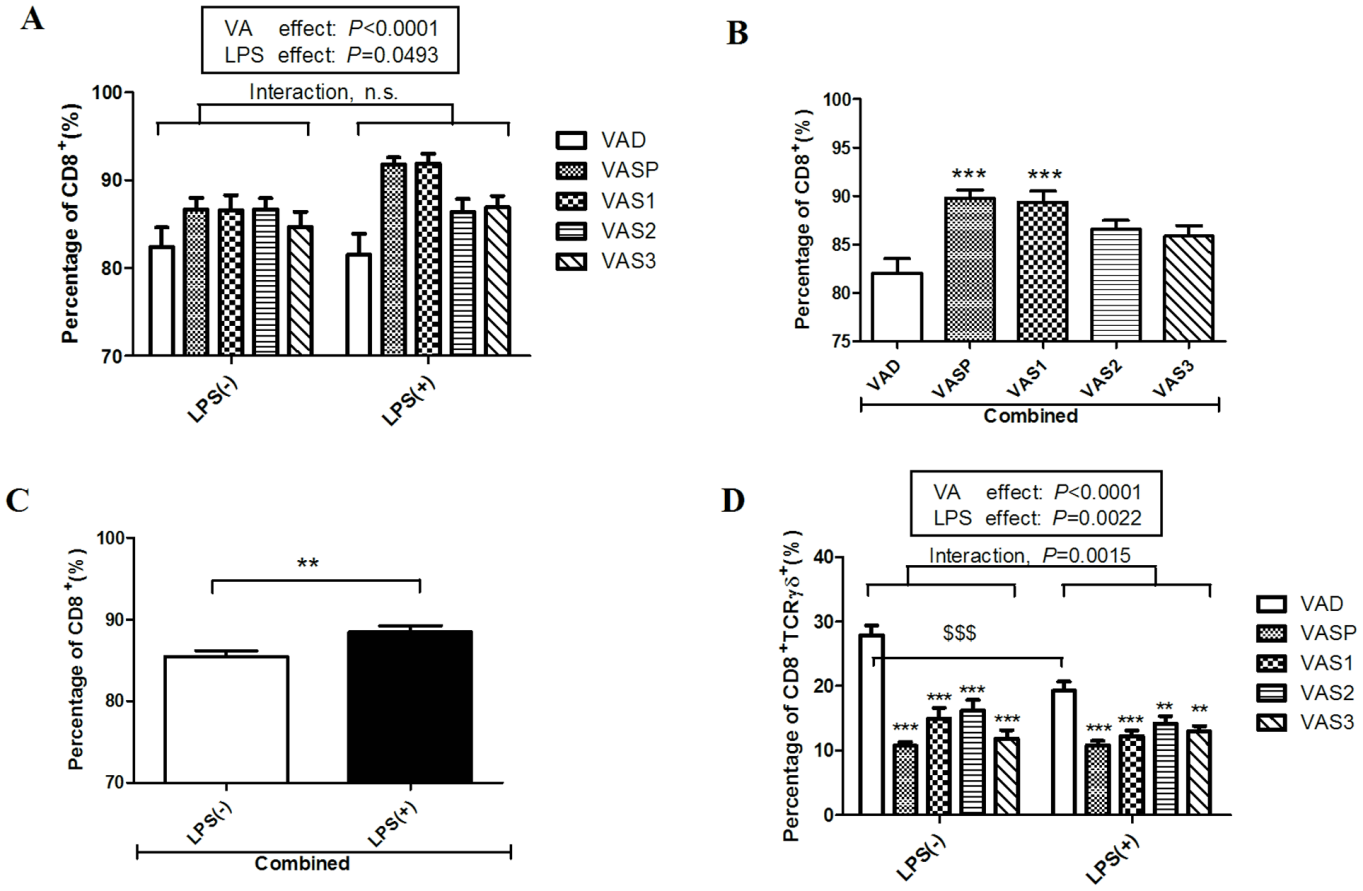
Changes in the percentage of CD8^+^ and CD8^+^TCRγδ^+^ IELs in the rats in the VAD group and in the rats in the four VAS groups in the presence or absence of LPS (n = 8 to 10). A) The effects of VAS and LPS challenge on the percentage of CD8^+^ IELs in the rats in the five groups (VAD, VASP, VAS1, VAS2 and VAS3). B) The number of CD8^+^ IELs was significantly higher in the rats in the combined VASP and VAS1 groups compared with the rats in the VAD group. C) The LPS treatment significantly affected the percentage of CD8^+^ IELs after combining data from the five VA treatments. D) The percentages of CD8^+^TCRγδ^+^ IELs were significantly decreased in the rats in the four VAS groups independent of exposure to LPS (n = 8 to 10). The values are the means±SD, and **P<0.01, ***P<0.001, ^$$$^ *P*<0.001; n.s. = not significant in post hoc tests.

IELs represent the first line of defense against microbes. In our previous study, VAD in early life decreased the number of CD8^+^ IELs. Thus, we further explored whether VAS increased the percentage of intestinal intraepithelial T lymphocytes that were CD8^+^ IELs and CD8^+^ TCRγδ^+^ IELs in the presence or absence of LPS. As Fig. 6A shows, the interaction between VAS and LPS challenge had no effect on CD8^+^ IELs; however, both VA (*P*<0.0001) and LPS (*P* = 0.0493) had significant independent effects on CD8^+^ IELs. In VAS rats, the percentage of CD8^+^ IELs was significantly higher in the rats in the combined VASP and VAS1 groups than in the rats in the combined VAD group (Fig. 6B). Furthermore, the LPS challenge, which was independent of the VA effect, significantly elevated the CD8^+^ IELs counts (*P*<0.01; Fig. 6C). The interaction between VAS and LPS treatments (*P* = 0.0015) had a significant effect on the percentage of CD8^+^TCRγδ^+^ IELs (Fig. 6D). The numbers of CD8^+^TCRγδ^+^ IELs were significantly lower in the rats in the VAD+LPS group compared with the rats in the VAD LPS-null group (*P*<0.001). The CD8^+^TCRγδ^+^ IEL numbers were significantly lower in the rats in the four VAS groups compared with the rats in the VAD group (Fig. 6D). Taken together, these results demonstrate that VAS during gestation and early postnatal life plays a critical role in promoting the mucosal immune response by increasing the number of CD8^+^ IELs.

### Vitamin A supplementation on postnatal day 1 enhanced the level of intestinal pIgR mRNA expression following LPS challenge

**Figure 7:**
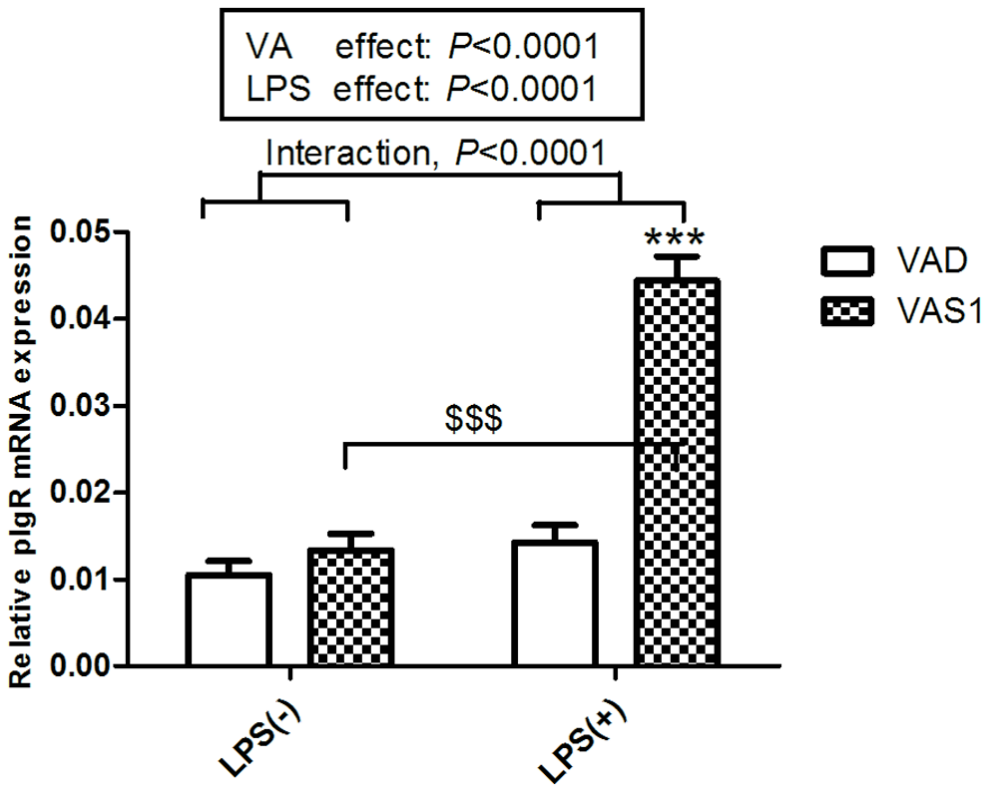
Effects of VAS and LPS challenge on the levels of pIgR mRNA expression in the intestine. The changes in pIgR mRNA expression levels in the intestine were determined in both the VAS1 and VAD rats with or without LPS challenge using real-time PCR (n = 8). The values are the means±SD, ***P<0.001, ^$$$^ *P*<0.001; and interaction *P*<0.0001 in post hoc tests.

pIgR is inextricably involved in the mucosal immune system of the intestine because of its essential role in the transport of sIgA into external secretion. Based on the above finding that VAS on postnatal day 1 can effectively improve the number of immune cells, we examined the effect of VAS on the level of pIgR mRNA expression in the VAS1 rats. Fig. 7 showed that the level of pIgR mRNA expression was significantly higher in the VAS1 rats compared with the VAD rats in the presence of LPS (*P*<0.001). Moreover, the mRNA level of pIgR was markedly induced in the LPS-challenged VAS1 rats (*P*<0.001) compared with the LPS-null treatment group. The post hoc test showed that the expression levels of pIgR mRNA were significantly affected by both the VA treatment (*P*<0.0001) and LPS challenge (*P*<0.0001) and that there was an interaction between the VA level and LPS challenge (*P*<0.0001; Fig. 7), which is consistent with the effects on the IgA levels of the intestine.

## Discussion

The aim of the present study was to clarify whether VAS could replenish the pool of immune cells depleted by gestational VAD. The primary finding of this study was that the strongest activation of the mucosal immune response occurred with VAS on postnatal day 1.

VA plays a critical role in innate immunity and in both cell-mediated and humoral antibody immunity [25]. A meta-analysis suggested that VAS reduced the risk of mortality and the incidence of diarrhea in children [12]; however, the optimal time point for improving intestinal mucosal immunity with VAS was previously unknown. In the current study, pregnant VAD rats or their VAD offspring received a low-dose of vitamin A daily for 7 days [19]. We found that the levels of serum retinol in the pups reached more than 1.05 µmol/L in all four VAS groups in the presence or absence of LPS challenge (Fig. 2A), indicating that VAS administered to VAD rats successfully increased serum retinol levels. Moreover, the LPS challenge produced a noticeable decrease in retinol levels in the rats in the four VAS groups (Fig. 2A). VA is an antioxidant vitamin that plays an important anti-inflammatory role. LPS challenge causes infection responses that decrease serum retinol levels [26]. Our study indirectly demonstrated that the LPS treatment was effective. However, the effects of the interaction between VA and LPS on serum retinol levels were not statistically significant.

sIgA contributes to the maintenance of the intestinal barrier, prevents infection and is involved in immunological homeostasis [27]. Our current study showed that IgA levels were markedly increased in the rats in the VASP, VAS1 and VAS2 groups following LPS challenge (Fig. 2D), which suggests that VAS in early life effectively stimulates the innate immune system in the intestine by increasing IgA secretion. This finding is consistent with the results of a recent study showing that IgA levels were increased by the oral administration of RA [28]. In addition to the function of sIgA, the significantly high expression of pIgR in VAS1 rats upon LPS challenge suggests that VAS on postnatal day 1 is required for the up-regulation of pIgR to increase the transport of sIgA in response to mucosal infection. In the present study, sIgA and pIgR levels were affected by both VAS and LPS. However, the interaction between VA and LPS also had a significant impact on the levels of sIgA and pIgR (Fig. 2D, Fig. 7).

Intestinal homeostasis is achieved by the actions of many cell populations via a set of shared network pathways. CD4^+^CD25^+^ T cells are T lymphocytes that regulate immune activation and maintain immune homeostasis. Although VAS had a significant effect on the percentage of CD4^+^CD25^+^ T cells in the spleen, this percentage increased significantly only in the VAS1 group (Fig. 3B), which suggests that VA intervention in early life (particularly on postnatal day 1) represents a unique “therapeutic window” during which the number of splenic CD4^+^CD25^+^ T cells in the offspring of gestational VAD rats can be restored. A study by Mucida demonstrated that RA could promote the differentiation of native CD4^ +^ T cells into inducible regulatory T cells and suppress the proliferation of Th17 cells [29]. The LPS challenge induced a significant increase in the number of CD4^+^CD25^+^ T cells in the spleen independent of the VAS effect, which further confirms that an intraperitoneal injection of LPS can activate systemic immune responses by up-regulating the number of splenic CD4^+^CD25^+^ T cells (Fig. 3C). CD4^+^CD8^+^ T cells are a type of antigen-specific memory cell that have an immune function similar to that of NK cells [30]. Although VAD statistically decreased the percentage of CD4^+^CD8^+^ T cells in the spleen in our previous study [10], VAS did not significantly increase the number of CD4^+^CD8^+^ T cells in the spleen in the present study (Fig. 3E). These data imply that VAS could not counteract the decrease in splenic CD4^+^CD8^+^ T cells caused by gestational VAD. However, the LPS challenge reduced the number of CD4^+^CD8^+^ T cells (Fig. 3F) independent of VAS.

Interestingly, gestational VAD increased the percentage of B lymphocytes in the MLNs in our previous study [10]; however, in the present study, VAS during the last gestational stage and on postnatal day 1 significantly decreased the number of B cells in the MLNs (Fig. 4A, 4B). RA is critical for intestinal mucosal immunity; it up-regulates the expression of gut-homing receptors (integrin α4β7 and CCR9) that enhance the homing and secreting ability of IgA in the B cells of the small intestine [31]. Thus, we propose that VA intervention in early life could directly promote the homing of B cells to the gut from the MLNs, thereby inducing the secretion of IgA in the intestine following LPS challenge.

To better understand some of the potential effects of VAS that involve the intestinal mucosal immune response, we further studied the local immune cells in the PPs and IELs. RA is synthesized locally in intestinal DCs that up-regulate the levels of α4β7 and CCR9 to promote T and B cell activation and migration to the intestine [31], [32]. Rodents with a VA-deficient diet experienced a dramatic loss of DCs in the PPs and a failure to induce gut-homing receptor expression [33]. The data obtained from the present study indicate that VAS on postnatal day 1 can increase the percentage of CD11c^+^ DCs within the PPs (Fig. 5B); while no differences in the number of CD4^+^CD25^+^ T cells were found between the rats in the four VAS groups and the VAD group, the percentages of CD4^+^CD25^+^ T cells in the VASP and VAS1 groups tended to increase (Fig. 5D). Therefore, to better elicit the mucosal immune response in the intestine, oral administration of LPS or other serotypes of LPS will be used in a future study.

IELs are effector T lymphocytes involved in the mucosal immune response of the intestine, which contains large quantities of CD8^+^ and TCRγδ^+^ T lymphocytes [34]. The CD8^+^ IELs display cytotoxic characteristics, whereas TCRγδ^+^ IELs are similar to NK cells [35]. The post hoc test showed that VAS during both the late-gestational period and on postnatal day 1 increased the numbers of CD8^+^ IELs (Fig. 6B). The LPS challenge also increased the number of CD8^+^ IELs (Fig. 6C), which suggests that the immunological activity of CD8^+^ IELs was affected by either VAS in early life or by the LPS treatment. However, the proportion of TCRγδ^+^ IELs was significantly reduced by VAS (Fig. 6D). This finding was consistent with the results of our previous study [10]. RA inhibited the expansion and activation of TCRγδ^+^ T cells, which decreased the regulation of Th17 cells [36]. An intraperitoneal injection of LPS induced a significant recruitment of TCRγδ T lymphocytes to the peritoneal cavity, which led to a decrease in the number of IELs [37]. The biological functions of VA during the development of IELs are poorly understood; however, it appears that VAS can affect the development of TCRγδ^+^ IELs and further regulate their immunomodulatory function. The findings from the present study suggest that both VAS and exposure to LPS enhance the cytotoxicity of IELs.

## Conclusions

The present study demonstrates that VAS in early life improves the systemic and intestinal mucosal immune function by increasing the percentage of CD8^+^ IELs and CD11_C_^+^ DCs in the PPs, the percentage of CD4^+^CD25^+^ T cells in the spleen and the levels of intestinal IgA. VAS also decreases the percentage of B cells in the MLNs and the number of TCRγδ^+^ IELs. Interactions between VAS and exposure to LPS significantly affected the levels of IgA secretion and pIgR expression, the percentage of TCRγδ^+^ IELs. These results provide novel evidence that the early-life stage represents the critical therapeutic window during which VAS can improve intestinal mucosal immune dysfunction caused by gestational VAD.
